# Impact of *Moringa oleifera* on rumen fermentation and methane emission under in vitro condition

**DOI:** 10.1186/s13568-022-01480-0

**Published:** 2022-11-12

**Authors:** Vandana Kumari Leitanthem, Parul Chaudhary, Mukesh Bhakat, Madhu Mohini, Goutam Mondal

**Affiliations:** 1 Animal Nutrition Division, ICAR-National Dairy Research Institute, Karnal, 132001 Haryana India; 2 Livestock Production and Management Division, ICAR-National Dairy Research Institute, Karnal 132001 Haryana, India

**Keywords:** *Moringa oleifera*, Methane emission, Digestibility, Rumen fermentation

## Abstract

Exploring innovative methods to provide essential nutrients and reducing ruminant greenhouse gas emission is crucial for animal production and diminishing global warming. This study was conducted to examine the efficacy of *Moringa oleifera* leaves (ML) in ruminants at 0%, 5%, 10%, 15%, 20%, 30% and 40% level in different roughage (R) and concentrate (C) (80R:20C, 70R:30C and 60R:40C) under in vitro conditions. Chemical composition of ML, concentrate mixture and berseem were estimated. Rumen fermentation parameters of male goat kids viz., total gas production, CH4, true dry matter digestibility (TDMD), organic matter digestibility (TOMD), partial fraction (PF), microbial biomass (MBP), ammonia (N), acetate, propionate, butyrate and acetate propionate ratio were observed under in vitro conditions. Results revealed that crude protein, organic matter and ethyl ether content were higher in ML as compared to concentrate mixture and berseem. Magnesium and iron content were also higher in ML as compared to concentrate and berseem. Total gas production, digestibility of DM and OM, MBP, acetate and propionate level were improved (*P* < 0.05) upto 10–20% replacement. In contrast, decreased in CH_4_ (%) and CH_4_ (mL/100 mg dDM) was noted with increased levels of ML incorporation. There was no change observed in ammonia, acetate: propionate ratios at all the three planes of nutrition. In this study, it is concluded that mixing *Moringa oleifera* leaves in feed can be used as protein supplement and reduce the methane emission without causing any effect on digestibility and rumen fermentation parameters. However, ML can be suggested for widespread practice to attain the sustainable animal production (10–20%) and to alleviate the global warming.

## Introduction

Livestock rearing is important for global food production. Animal production in farm is typically reduced due to the low quality and scarcity of animal feeds in tropical countries. Feedstuffs especially protein sources such as legumes, cereals and grains essential for animal development, have become very expensive and limited in many regions of the world (Choudhary et al. 2022). Hence, it is required for searching a substitute source of feed which are edible, rich in protein and minerals, low-priced and fulfils the basic needs of small ruminants.

The ruminant cattle industry significantly contributes to greenhouse gas (GHG) emission that cause global warming (Eisen and Brown 2022). Methane is one of the main GHG and its potency is twenty-five times as that of CO_2_. Ruminants are one major causes of emission of methane (81–92 MT) produced per year worldwide which is equal to total anthropogenic methane (23–27%). Methane is produced by enteric fermentation process in ruminants and contributes about 13% of methane emission from livestock in India (Gupta et al. 2018). Cattle contribute 49.10% enteric methane emission followed by buffalo, goat, sheep and others as 42.80%, 5.38%, 2.59% and 0.73% within agriculture. Different sources such as amino acids, organic acids, essential oils and exogenous enzymes have been used to alleviate the ruminant methane emission (Benetal et al. 2022; Kholif et al. 2022). Numerous studies have reported a reduction in enteric methane emission by feeding of tree leaves to ruminants and many workers have advocated their use as an alternative protein source for livestock (Ku-Vera et al. 2020).

*Moringa oleifera* is a perennial tree feed and also called as “miracle tree”. It is a multipurpose and fast-growing tree with nutritional and therapeutic properties that can be planted in a variety of climates including drought and heat, and can be harvested numerous times (Abbas et al. 2018). Its leaves contain sufficient quantity of minerals, proteins and vitamins according to the nutritional demand in lactating goats (Afzal et al. 2022). It is also having antioxidant properties such phenols, vitamin C, carotene and flavonoids (Saleem et al. 2020). It is an inexpensive protein constituent as compared to soyabean and sesame feed meals used in livestock feeding. *Moringa oleifera* leaves (ML) meal contains 9 times extra protein as compared to yogurt having good feeding effect and can be used as protein substitute in animal feed (Su and Chen 2020). The normal crude protein content in ML was 180–270 g CP/kg DM as reported by Kholif et al. (2018). Application of *Moringa* foliage improved the feed consumption, metabolic profile and growth performance of goat kids (Wankhede et al. 2022). ML are natural feed supplement which produce secondary metabolites like tannins and saponins, modify the pathways of rumen fermentation and prevent the growth of methanogens effectively (Zeru et al. 2022). ML strengthened the immune system and reduced oxidative stress in goats due to the presence of bioactive compounds (Al-Juhaimi et al. 2020; Teclegeorgish et al. 2021). Dong et al. (2019) reported that supplementation of ML in goats food improved content of fat milk and decrease the *M. ruminantium* which involved in methane production. Application of *Moringa* oil (4%) along with 30–50% of roughage ration decreased the methanogens and protozoa population but increase the *Provotella* which involved in rumen acidosis (Ebeid et al. 2020). There has been less research on the effects of ML at various concentration on male goat kids to reduce enteric methane emission, and how it affects rumen fermentation kinetics and digestibility. Hence, the purpose of this study was to investigate the consequence of *M. oleifera* leaves on rumen fermentation and methane production under in vitro conditions with different plane of nutrition.

## Materials and methods

### Farm description and feed preparation

The present study was conducted in Animal Nutrition Division, National Dairy Research Institute (NDRI), Karnal, Haryana, India. This institution is located at 29° 42ʹN and 79° 54ʹE at 834 feet above the sea level. *Moringa oleifera* leaves was collected from NDRI farm, shade and oven dried, grind and powder were packed in airtight polythene bags, while berseem and concentrate mixture were oven dried at 60 ℃. Desiccated samples were crushed and sieve (1 mm) by using electrically operated Wiley mill. After complete drying, sample was grinded and placed in sample bottles for further use. Dried samples were used for analysis of DM, OM, CP and EE as per AOAC (2005), NDF and ADF (Van Soest et al. 1991).

### Estimation of secondary metabolites in *Moringa oleifera* leaves

Tannin estimation was carried out using the method given by Nwinuka et al. (2005). Tannic acid (1 mg/mL) was used as the reference. Plant leaves extract (1 mL) was mixed with Folin-Ciocalteu reagent (0.5 mL) and sodium carbonate solution (1 mL). The total volume was made up to 5 mL. Tannins concentration was determined by measuring absorbance at 755 nm.

Methanolic extract of leaves (500 µL) and anisaldehyde reagent (500 µL, 0.5%) were mixed in a test tube and left for 10 min for saponins estimation. Sulphuric acid (5%, 2 mL) was added in tubes, mixed properly and kept in water bath at 60℃ for 10 min. The tubes were cooled and the absorbance was measured at 435 nm (Baccou et al. 1977).

Total phenolic content (TPC) in methanolic extract of ML was determined using the Folin-Ciocalteu reagent assay. Folin-Ciocalteu (750 mL), sodium carbonate (7.5%, 2 mL) and leaves methanolic extract (200 ml) were added in a tube. The mixture was diluted with deionized water to 7 mL, then left at room temperature in the dark for 2 h. The absorbance was measured at 765 nm using spectrophotometer **(**Folin and Ciocalteu 1927).

The total flavonoid content (TFC) was determined using the method described by Zhishen et al. (1999). Briefly, methanolic extract (1 mL) was added to a 10 mL volumetric flask containing water (4 mL). Sodium nitrate (0.3 mL, 5%) was added in flask followed by aluminum chloride (0.3 mL, 10%) at 5 min, sodium hydroxide (2 mL, 1 M) at 6 min. Then 2.4 mL water was added in flask and absorbance was measured at 510 nm.

### Mineral composition of feed ingredients

Dried (0.5 g) dried and crushed feed sample was weighted into digestion tubes, tri acid mixture (10 mL) was added for digestion in Kelplus micro digestion assembly. The absence of white fumes and black particles in the residues suggested that samples had been completely digested. The digested samples were then filtered using filter paper. Filter paper was rinsed many times with double distilled water and Inductivity coupled plasma (ICP-Optical Emission Spectrometer was used for mineral analysis, while P was estimated using spectrophotometric method (Sultana 2020).

### Experimental design and rumen liquor collection

Rumen liquor was collected from the male goat kids with stomach tube and vaccum pump fitted with sterile pipe connected to it in early morning before feeding in pre-warmed thermos flask, filtered using four layers of muslin cloth and used as an inoculum for in vitro gas production in different treatments **(**Menke and Steingass 1988**)**. Details of the experiment as follows: ML at different concentration (0, 5, 10, 15, 20, 30 and 40% replaced with concentrate mixture) along with three different ratios of roughage and compound feed (60R:40C, 70R:30C and 80R:20C) were prepared for in vitro experiment. This trial was conducted with three replications with 3 sub replicates of each treatment (total n = 9 samples). Different substrates (200 mg) were weighted separately and added in calibrated glass syringes (100 mL) at the bottom side. The piston was greased through petroleum jelly and hard-pressed inside the syringe. These syringes were incubated at 39 ℃ in water bath. Solution media was prepared separately which contains micro mineral solution (0.124 mL), macro mineral solution (250 mL), rumen buffer solution (250 mL), resazurin solution (1.25 mL) and distilled water (500 mL). This solution was prewarmed at 39 ℃ and fizzed with CO_2,_ when this mixture become colorless rumen liquor was added. After proper mixing this mixture (30 mL) was injected in syringes through auto dispenser, shaken gently, outlet closed and incubated in water bath for 24 h at 39 ℃ for further use. Piston level was checked for initial reading and syringes were shaken after every 30 min.

#### Analysis of total gas production

After 24 h incubation of the above mixture, total gas production was estimated by deducting the initial reading as of final reading.

#### Estimation of in vitro methane production

The in vitro methane (CH_4_) production was estimated by taking the sample gas after 24 h of incubation from the head space in the air tight syringe and injected to gas chromatogram fitted with flame ionization detector and stainless-steel column packed with Porapak-Q (1.5 m × 3.2 mm × 2 mm). The temperature for injector column and detector were adjusted as 40, 50 and 100 ℃ and flow rate for nitrogen gas through column was 30, 300 and 30 mL/min. Gas sample (2 mL) from syringe was injected into GC through injection port. The standard gas used for methane estimation composed of methane and CO2 (50%).

#### Estimation of true dry and organic matter digestibility (TDMD/TOMD), partial fraction (PF) and microbial biomass (MBN)

After 24 h incubation, syringe samples from each replicate were transferred to centrifuge tubes and centrifuged for 10 min at 3000 rpm. Obtained pellet was used for the estimation of DM and OM. Pellets obtained after centrifugation were refluxed with neutral detergent solution, filtered using G1 crucibles and then residues were oven dried to evaluate the TOMD. For in vitro organic matter digestibility (IVTOMD) estimation the residue was ashed at 550–650 ℃ in Muffle furnace. Microbial biomass production and partial fraction were estimated using TDOM (Blummel et al. 1997).

#### Estimation of ammonia production

Aliquot gas sample was used for the assessment of ammonia nitrogen (NH_3_–N) by Kjeldahl process. Supernatant was acidified with equal volume of HCl (0.5 M) and placed at − 20 ℃. Acidified supernatant (5 mL) and NaOH (10 mL) steam distilled using KELPLUS-N analyzer (Pelican, India). The ammonia was collected in boric acid solution (20%) comprising of mixed indicators and then titrated with H_2_SO_4_ (Komolong et al. 2001).

#### Estimation of volatile fatty acids (VFA)

Supernatant (4 mL) was added in m-phosphoric acid (25%, 2 mL) and overnight placed at 4 ℃. The solution was centrifuged for 15 min at 3000 rpm and stored at 4 ℃ for VFA analysis. The individual VFA in different samples were estimated using gas chromatograph (Nucon 5700) equipped with flame ionization detector and stainless-steel column packed with chromosorb 101. Analytical conditions used for fractionation of VFA was injector port temperature (210 ℃), column temperature (180 ℃), detector temperature (230 ℃). Flow rate of carrier nitrogen gas was 40 mL/min **(**Bannink et al. 2006).

### Statistical analysis

Results were statistically analyzed using SPSS software ver. 16.0 through one-way analysis of variance at *P* < 0.05. The values of above parameters were presented as mean ± standard error.

## Results

### Chemical composition of feed ingredients and total mixed ration (TMR)

**Fig. 1:**
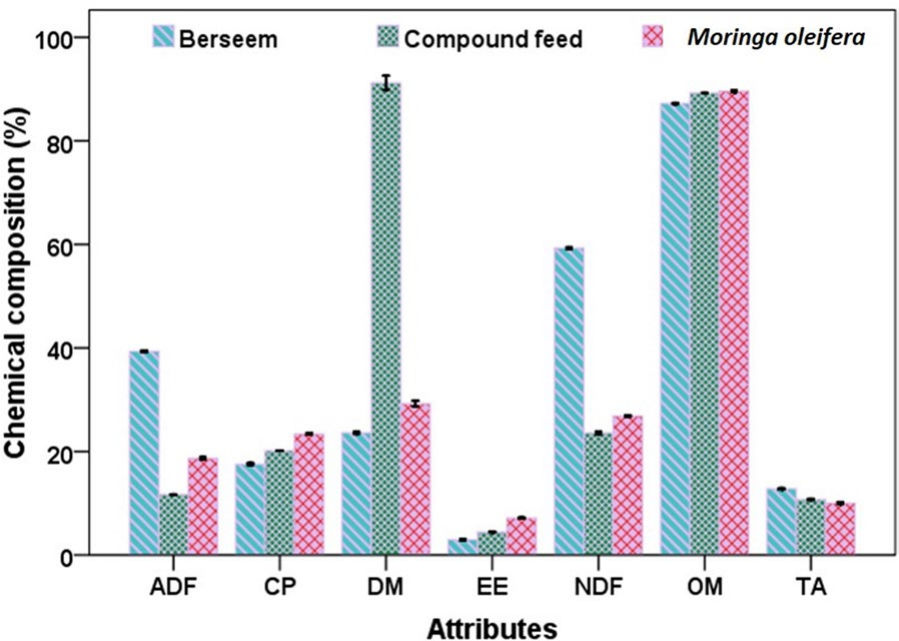
Chemical composition of berseem, compound feed and *Moringa oleifera*

The chemical composition of berseem fodder, concentrate and *M. oleifera* leaves are presented in the Fig. 1. DM content was higher in concentrate/compound feed (91.21%) followed by *M. oleifera* (29.26%) and berseem (23.61%). Organic matter, CP and EE content were higher in *M. oleifera* leaves (89.62, 23.37 and 7.13%) followed by compound feed (89.26, 20.14, 4.45%) and berseem (87.22, 17.56 and 2.98%), respectively. The content of NDF, ADF and TA were 59.29, 39.33 and 12.78% in berseem, 23.58, 11.69, 10.74% in compound feed and 26.86, 18.70 and 10.03% in *M. oleifera,* respectively.

The chemical composition of TMR is presented in Table 1. The CP (%) in 60R:40C was higher in 40% TMR (19.11) followed by 18.98 in 30%, 18.85 in 20%, 18.79% in 15% TMR, 18.72% in 10%, 18.66% in 5% and 18.59 in 0% TMR. In 70R:30C group, CP (%) was higher in 40% TMR (19.74%) followed by 18.86 in 20%, 18.62 in 30%, 18.48% in 15% TMR, 18.43% in 10%, 18.38% in 5% and 18.33 in 0% TMR. The CP (%) in 80R:20C group was higher in 40% TMR (18.33%) followed by 18.27 in 30%, 18.21 in 20%, 18.17% in 15% TMR, 18.14% in 10% and 0% and 18.11% in 5% TMR.

**Table 1:** Chemical composition (%) of total mixture ration in different groups (60R:40C, 70R:30C and 80R:20C)

Attributes (%)	0%	5%	10%	15%	20%	30%	40%
DM (60R:40C)	50.65 ± 0.35	49.41 ± 0.48	48.17 ± 0.53	46.93 ± 0.43	45.69 ± 0.30	43.22 ± 0.58	40.74 ± 0.52
OM	88.04 ± 0.15	88.04 ± 0.13	88.05 ± 0.25	88.06 ± 0.36	88.06 ± 0.26	88.08 ± 0.32	88.09 ± 0.20
CP	18.59 ± 0.53	18.66 ± 0.33	18.72 ± 0.27	18.79 ± 0.29	18.85 ± 0.15	18.98 ± 0.28	19.11 ± 0.11
EE	3.57 ± 0.21	3.62 ± 0.15	3.68 ± 0.12	3.73 ± 0.11	3.78 ± 0.10	3.89 ± 0.20	4.00 ± 0.12
NDF	45.01 ± 0.22	45.07 ± 0.44	45.14 ± 0.25	45.20 ± 0.42	45.27 ± 0.52	45.40 ± 0.40	45.53 ± 0.43
ADF	28.27 ± 0.28	28.41 ± 0.41	28.55 ± 0.35	28.69 ± 0.40	28.83 ± 0.27	29.12 ± 0.13	29.40 ± 0.40
TA	11.96 ± 0.08	11.95 ± 0.16	11.94 ± 0.18	11.92 ± 0.07	11.91 ± 0.21	11.88 ± 0.11	11.85 ± 0.15
DM (70R:30C)	43.89 ± 0.35	42.96 ± 0.44	42.03 ± 0.47	41.10 ± 0.54	39.41 ± 0.41	38.31 ± 0.58	45.58 ± 0.59
OM	87.83 ± 0.74	87.84 ± 0.40	87.84 ± 0.44	87.85 ± 0.63	86.14 ± 0.58	87.86 ± 0.54	96.80 ± 0.68
CP	18.33 ± 0.33	18.38 ± 0.38	18.43 ± 0.34	18.48 ± 0.28	18.86 ± 0.36	18.62 ± 0.37	19.74 ± 0.26
EE	3.42 ± 0.07	3.46 ± 0.09	3.50 ± 0.09	3.54 ± 0.05	3.28 ± 0.06	3.66 ± 0.06	4.19 ± 0.12
NDF	48.58 ± 0.53	48.63 ± 0.36	48.68 ± 0.31	48.72 ± 0.49	49.65 ± 0.46	48.86 ± 0.51	51.33 ± 0.55
ADF	31.04 ± 0.75	31.14 ± 0.60	31.25 ± 0.75	31.35 ± 0.68	31.99 ± 0.32	31.67 ± 0.77	33.05 ± 0.70
TA	12.17 ± 0.07	12.16 ± 0.16	12.15 ± 0.15	12.14 ± 0.07	12.06 ± 0.08	12.10 ± 0.10	13.16 ± 0.10
DM (80R:20C)	35.89 ± 0.50	36.51 ± 0.48	35.89 ± 0.60	35.27 ± 0.42	34.65 ± 0.65	33.41 ± 0.41	32.17 ± 0.64
OM	87.64 ± 0.64	87.63 ± 0.63	87.64 ± 0.35	87.64 ± 0.35	87.64 ± 0.38	87.65 ± 0.45	87.66 ± 0.51
CP	18.14 ± 0.14	18.11 ± 0.12	18.14 ± 0.06	18.17 ± 0.14	18.21 ± 0.12	18.27 ± 0.15	18.33 ± 0.12
EE	3.33 ± 0.11	3.30 ± 0.07	3.33 ± 0.11	3.35 ± 0.07	3.38 ± 0.04	3.43 ± 0.05	3.49 ± 0.06
NDF	52.21 ± 0.29	52.18 ± 0.18	52.21 ± 0.21	52.25 ± 0.54	52.28 ± 0.30	52.34 ± 0.45	52.41 ± 0.52
ADF	33.94 ± 0.53	33.87 ± 0.27	33.94 ± 0.74	34.01 ± 0.31	34.08 ± 0.65	34.22 ± 0.58	34.36 ± 0.63
TA	12.36 ± 0.10	12.36 ± 0.12	12.36 ± 0.14	12.35 ± 0.05	12.34 ± 0.05	12.33 ± 0.05	12.32 ± 0.10

### Secondary metabolites in *Moringa oleifera* leaves

On the basis of % dry matter, the value of each chemical component such as tannin, saponin, TPC and TFC in *M. oleifera* leaves was calculated. The TPC content was higher (4.28%) followed by TFC (3.61%), tannin (2.02%) and saponin (1.01%), respectively.

### Mineral composition of berseem fodder, concentrate and *Moringa oleifera *leaves

Calcium content was higher in berseem (1.92%) followed by *M. oleifera* (1.85%) and concentrate mixture (1.37%), while phosphorus content was higher in concentrate mixture (0.79%) followed by berseem fodder (0.38%) and *M. oleifera* (0.15%). Magnesium content was higher in *M. oleifera* (4.81%) followed by concentrate mixture (0.81%) and berseem fodder (0.40%). The Fe (ppm) content was 287 in concentrate mixture, berseem fodder (289 ppm) and *M. oleifera* (330 ppm). The level of Cu and Zn (ppm) in concentrate mixture (29.21, 49), berseem fodder (4.61, 16.41) and *M. oleifera* (9.21 and 26.72 ppm) presented in Table 2.

**Table 2:** Proximate mineral composition of berseem fodder, concentrate and Moringa leaves

Parameter	Concentrate mixture	Berseem fodder	*Moringa oleifera*
Ca (%)	1.37 ± 0.05	1.92 ± 0.02	1.85 ± 0.12
P (%)	0.79 ± 0.10	0.38 ± 0.04	0.15 ± 0.05
Mg (%)	0.81 ± 0.03	0.40 ± 0.03	4.81 ± 0.14
Fe (ppm)	287.00 ± 0.50	289.53 ± 0.65	330 ± 0.33
Cu (ppm)	29.21 ± 0.23	4.61 ± 0.06	9.21 ± 0.23
Zn (ppm)	49.00 ± 0.45	16.41 ± 0.23	26.72 ± 0.31

### Fermentation parameters

The total gas production increased (*P* < 0.05) at 10% replacement of concentrate feed in 60R:40C group. The values for total gas production in 60R:40C was 162.93 and 168.34 (mL/gDM) at 10% and 15% level, no further difference was observed after these replacement level (Table 3). IVDMD, IVOMD, acetate, MBN production were higher at 15% replacement in 60R:40C, while propionate was higher at 10% replacement level (*P* > 0.05) than other replacement groups. It was observed that with increased replacement of concentrate with ML, there was significant reduction (*P* < 0.05) in CH4 (%) and CH4 ml/100 mg of digestible dry matter (dDM). The CH_4_ (%) and CH_4_ (mL/100 mg) in 60R:40C were 30.22 and 7.50 at 10% level of replacement. Reduction in methane was observed at 10% replacement, however no further reduction (*P* < 0.05) in methane was observed above this level. A significant (*P* < 0.05) increase in the acetate (57.90% and 59.81%) and propionate (19.42% and 19.73%) production was observed upto 10% and 15% replacement in 60R:40C. The ammonia nitrogen (mg/dL) was similar (*P* < 0.05) in all planes of nutrition. The values for ammonia-N (mg/dL) in 60R:40C was 13.14 at 0%, 12.64 at 5%, 13.08 at 10%, 12.14 at 15%, 14.17 at 20%, 12.69 at 30% and 12.02 at 40% level, respectively. The values for partition factor in 60R:40C was 4.04 at 0%, 3.94 at 5%, 4.01 at 10%, 3.88 at 15%, 3.85 at 20%, 4.20 at 30% and 5.24 at 40% level, respectively. There was an increase (*P* < 0.05) in MBP (mg) at 15% level as compared other level (Table 3).

**Table 3:** Effect of *Moringa oleifera* leaf on in vitro digestibility, total gas, methane, PF and MBP along with roughage and concentrate feed (60R:40C)

Parameters	0%	5%	10%	15%	20%	30%	40%	SEM	P value
Total gas (mL/gDM)	150.43 ± 3.89^c^	156.41 ± 4.76^bc^	162.93 ± 0.10^ab^	168.34 ± 4.88^ab^	173.29 ± 0.07^a^	171.15 ± 5.23^a^	171.65 ± 0.24^a^	2.274	0.020
IVTDMD (%)	68.83 ± 0.43^c^	70.06 ± 0.47^c^	74.12 ± 0.18^b^	75.20 ± 1.35^ab^	75.42 ± 0.52^ab^	77.92 ± 1.93^a^	77.94 ± 0.22^a^	0.797	< 0.001
IVTOMD (%)	71.21 ± 0.40^c^	72.75 ± 0.39^bc^	76.38 ± 1.18^b^	76.36 ± 0.72^ab^	76.73 ± 0.65^ab^	78.54 ± 0.25^ab^	79.28 ± 1.74^a^	0.671	< 0.001
CH4 (%)	31.81 ± 0.14^a^	30.70 ± 0.23^b^	30.22 ± 0.25^b^	29.13 ± 0.35^c^	28.27 ± 0.30^d^	27.64 ± 0.31^d^	27.48 ± 0.25^d^	0.351	< 0.001
CH4 (mL/100 mg)	7.97 ± 0.14	7.78 ± 0.17	7.50 ± 0.37	7.34 ± 0.02	7.31 ± 0.52	7.03 ± 1.46	6.95 ± 0.26	0.341	0.99
Acetate(mM)	50.89 ± 0.73^d^	54.39 ± 1.31^cd^	57.90 ± 2.14^bc^	59.81 ± 2.58^abc^	62.60 ± 2.16^ab^	64.48 ± 3.26^ab^	65.50 ± 1.18^a^	1.292	0.002
Propionate (mM)	17.55 ± 0.67^c^	18.32 ± 0.99^bc^	19.42 ± 0.37^abc^	19.73 ± 0.69^abc^	20.13 ± 0.64^ab^	20.68 ± 1.21^ab^	21.18 ± 0.49^a^	0.360	0.05
Butyrate (mM)	10.08 ± 0.10	10.21 ± 0.72	11.35 ± 0.16	11.07 ± 0.51	10.33 ± 0.81	9.90 ± 0.78	9.42 ± 0.45	0.227	0.29
AP ratio	2.91 ± 0.12	2.98 ± 0.09	2.99 ± 0.14	3.03 ± 0.06	3.12 ± 0.19	3.12 ± 0.11	3.09 ± 0.02	0.040	0.79
PF	4.04 ± 0.06	3.94 ± 0.13	4.01 ± 0.23	3.88 ± 0.07	3.85 ± 0.33	4.20 ± 0.87	5.24 ± 2.27	0.308	0.936
MBP (mg)	49.91 ± 1.41^d^	52.31 ± 2.69^cd^	55.03 ± 1.07^bcd^	56.87 ± 1.91^abc^	58.61 ± 1.16^ab^	60.60 ± 0.36^ab^	61.52 ± 2.77^a^	1.056	0.004
Ammonia N (mg/dl)	13.14 ± 0.53	12.64 ± 0.84	13.08 ± 0.45	12.14 ± 1.33	14.17 ± 1.48	12.69 ± 1.47	12.02 ± 0.41	0.36	0.808

Means with different superscripts a, b, c and d in the same row differ significantly (*P* < 0.05)

The total gas production increased (*P* < 0.05) at 10% replacement of concentrate feed with ML in 70R:30C group. The total gas production in forage to concentrate 70R:30C ratio was 157.26(mL/gDM) at 10% level. The IVDMD (73.19%) and IVOMD (74.07%) production was higher at 20% replacement in 70R:30C than other level of replacement feed. The percentage of CH_4_ in 70R:30C was 32.16, 31.03, 30.55, 29.44, 28.57, 27.94 and 27.78% at 0, 5, 10, 15, 20, 30 and 40%, respectively. The values of CH_4_ (mL/100 mg dDM) were 8.12 at 0%, 7.80 at 5%, 7.87 at 10%, 7.44 at 15%, 6.90 at 20%, 6.79 at 30% and 6.88 at 40% level respectively. Reduction in methane level was observed upto 15–20% in 70R:30C. A significant (*P* < 0.05) increase in the acetate (%) and propionate (%) production were observed upto 20% replacement in 70R:30C and no further change was observed. No difference was observed in butyrate production and acetate butyrate ratios in all the groups at all levels of supplementation. Ammonia-N (mg/dL) in 70R:30C was 13.23 at 0%, 12.65 at 5%, 12.76 at 10%, 12.29 at 15%, 13.76 at 20%, 11.97 at 30% and 11.97 at 40% level respectively. The PF ratio in 70R:30C was 3.90 at 0%, 3.84 at 5%, 3.92 at 10%, 3.84 at 15%, 3.91 at 20%, 3.98 at 30% and 3.81 at 40% level, respectively (*P* > 0.05). There was significant increase (*P* < 0.05) in MBP (mg) at 20% level (53.17%) as compared to others, data presented in Table 4.

**Table 4:** Effect of replacement of *Moringa oleifera* leaf on in vitro digestibility, total gas, methane, PF and MBP along with roughage and concentrate feed (70R:30C)

Parameters	0%	5%	10%	15%	20%	30%	40%	SEM	P value
Total gas (mL/gDM)	146.43 ± 3.89^c^	151.41 ± 4.76^bc^	157.26 ± 0.80^abc^	163.68 ± 4.92^ab^	165.95 ± 4.62^ab^	168.48 ± 5.46^a^	171.32 ± 1.85^a^	2.42	0.02
IVTDMD (%)	62.64 ± 0.67^e^	64.25 ± 0.43^e^	66.56 ± 0.69^d^	69.58 ± 1.54^c^	73.19 ± 0.37^b^	74.52 ± 0.48^ab^	75.85 ± 0.16^a^	1.10	< 0.001
IVTOMD (%)	64.69 ± 0.14^c^	66.25 ± 0.40^c^	69.89 ± 1.31^b^	71.01 ± 1.46^b^	74.07 ± 0.81^a^	75.07 ± 0.47^a^	75.68 ± 0.01^a^	0.93	< 0.001
CH4 (%)	32.16 ± 0.15^a^	31.03 ± 0.24^b^	30.55 ± 0.25^b^	29.44 ± 0.35^c^	28.57 ± 0.30^d^	27.94 ± 0.31^d^	27.78 ± 0.25^d^	0.35	< 0.001
CH4 (mL/100 mg)	8.12 ± 0.13^a^	7.80 ± 0.15^ab^	7.87 ± 0.29^ab^	7.44 ± 0.31^bc^	6.90 ± 0.17^c^	6.79 ± 0.18^c^	6.88 ± 0.09^c^	0.13	0.001
Acetate (mM)	49.05 ± 1.23^c^	52.62 ± 0.68^bc^	56.83 ± 2.10^ab^	58.53 ± 2.46^ab^	61.01 ± 2.35^a^	62.98 ± 3.36^a^	63.47 ± 1.19^a^	1.30	0.002
Propionate (mM)	16.62 ± 0.52^c^	17.36 ± 1.22^bc^	18.69 ± 0.28^abc^	19.25 ± 0.86^ab^	19.66 ± 0.45^ab^	20.11 ± 1.19^a^	20.73 ± 0.38^a^	0.39	0.02
Butyrate (mM)	9.99 ± 0.30	10.04 ± 1.04	11.45 ± 0.16	10.87 ± 0.61	10.04 ± 0.91	9.14 ± 1.16	9.42 ± 0.53	0.29	0.41
AP ratio	2.96 ± 0.15	3.05 ± 0.17	3.04 ± 0.12	3.04 ± 0.04	3.11 ± 0.17	3.14 ± 0.11	3.06 ± 0.02	0.04	0.97
MBP (mg)	46.61 ± 0.46^d^	47.82 ± 1.91^cd^	49.94 ± 2.95^bcd^	50.17 ± 3.28^bcd^	53.17 ± 1.35^abc^	55.33 ± 1.27^ab^	56.85 ± 0.11^a^	1.005	0.018
PF	3.90 ± 0.17	3.84 ± 0.20	3.92 ± 0.06	3.84 ± 0.17	3.91 ± 0.13	3.98 ± 0.09	3.81 ± 0.09	0.047	
Ammonia N (mg/dl)	13.23 ± 0.48	12.65 ± 0.81	12.76 ± 0.83	12.29 ± 1.71	13.76 ± 0.56	11.97 ± 1.18	11.97 ± 0.49	0.33	0.821

Means with different superscripts a, b, c,  d and e in the same row differ significantly (*P* < 0.05)

The values for gas production in 80R:20C was 143.10, 151.08, 154.26, 163.68, 163.95, 166.15, 177.32 (mL/gDM) at 0, 5, 10, 15, 20, 30 and 40% level, respectively. The IVDMD (70.52%) and IVOMD (70.18%) production was higher at 20% and 15% replacement in 80R:20C than other level of feed. The percentage of CH_4_ in 80R:20C was 34.23, 33.03, 32.25, 30.53, 28.56, 27.94 and 26.78% at 0, 5, 10, 15, 20, 30 and 40% respectively. The values of CH_4_ (mL/100 mg dDM) in 80R:20C was 8.89 at 0%, 8.61 at 5%, 8.40 at 10%, 7.90 at 15%, 7.05 at 20%, 6.86 at 30% and 6.56 at 40% level respectively (Table 5). Reduction in methane level was observed up to 15–20% in 80R:20C. A significant (*P* < 0.05) increase in the acetate (55.76%) at 10% and propionate (19.37%) production were observed at 20% replacement in 80R:20C. No difference was observed in butyrate production and acetate butyrate ratios in all the groups at all levels of supplementation. Ammonia nitrogen (mg/dL) was similar (*P* < 0.05) in all the planes of nutrition. There was no significant increase in PF ration in different plane of nutrition. The PF ratio in 80R:20C was 2.90 at 0%, 2.84 at 5%, 2.92 at 10%, 2.84 at 15%, 2.91 at 20%, 2.98 at 30% and 2.81 at 40% level, respectively. There was significant increase (*P* < 0.05) in MBP (mg) at 15% level (51.34%) was observed as compared to other level. The values for MBP (mg) in 80R:20C was 42.65 at 0%, 45.38 at 5%, 47.63 at 10%, 51.34 at 15%, 53.28 at 20%, 54.43 at 30% and 56.82 at 40% level respectively.

**Table 5:** Effect of *Moringa oleifera* leaf on digestibility, total gas, methane, PF and MBP along with different roughage and concentrate feed (80R:20C)

Parameters	0%	5%	10%	15%	20%	30%	40%	SEM	P value
Total gas (mL/gDM)	143.10 ± 3.78^c^	151.08 ± 4.12^bc^	154.26 ± 1.95^abc^	163.68 ± 3.51^ab^	163.95 ± 3.89^ab^	166.15 ± 4.66^a^	167.32 ± 1.87^a^	2.307	0.01
IVTDMD (%)	60.31 ± 0.44^e^	61.59 ± 0.75^de^	63.90 ± 0.56^d^	66.58 ± 1.54^c^	70.52 ± 0.21^b^	71.85 ± 0.68^ab^	73.19 ± 0.37^a^	1.089	< 0.001
IVTOMD (%)	62.86 ± 0.70^d^	64.42 ± 0.07^d^	67.73 ± 1.01^c^	70.18 ± 0.21^b^	72.24 ± 0.48^ab^	74.24 ± 1.21^a^	73.85 ± 0.67^a^	0.962	< 0.001
CH4 (%)	34.23 ± 0.58^a^	33.03 ± 0.10^ab^	32.25 ± 0.21^b^	30.53 ± 0.85^c^	28.56 ± 0.57^d^	27.94 ± 0.31^de^	26.78 ± 0.17^e^	0.601	< 0.001
CH4 (mL/100 mg)	8.89^a^ ± 0.50^a^	8.61 ± 0.50^a^	8.40 ± 0.12^a^	7.90 ± 0.50^ab^	7.05 ± 0.31^bc^	6.86 ± 0.18^bc^	6.56 ± 0.11^c^	0.223	0.001
Acetate (mM)	47.92^d^ ± 1.24^d^	51.70 ± 0.20^cd^	55.76 ± 1.46^bc^	57.46 ± 1.98^ab^	57.23 ± 1.51^ab^	61.90 ± 2.89^a^	62.68 ± 1.54^a^	1.217	< 0.002
Propionate (mM)	16.11 ± 0.77^c^	16.80 ± 1.15^bc^	18.30 ± 0.33^ab^	18.79 ± 0.55^ab^	19.37 ± 0.39^a^	19.57 ± 0.87^a^	20.19 ± 0.07^a^	0.377	0.01
Butyrate (mM)	9.71 ± 0.43	9.58 ± 0.98	11.12 ± 0.07	10.31 ± 0.50	9.82 ± 0.76	8.97 ± 1.12	9.24 ± 0.48	0.267	0.45
AP ratio	2.99 ± 0.20	3.10 ± 0.20	3.05 ± 0.08	3.06 ± 0.04	2.95 ± 0.03	3.16 ± 0.09	3.10 ± 0.07	0.041	0.90
MBP (mg)	42.65 ± 1.25^d^	45.38 ± 0.95^cd^	47.63 ± 3.2^bcd^	51.34 ± 0.20^abc^	53.28 ± 2.59^ab^	54.43 ± 2.48^a^	56.82 ± 1.08^a^	1.234	0.001
PF	2.90 ± 0.17	2.84 ± 0.20	2.92 ± 0.06	2.84 ± 0.17	2.91 ± 0.13	2.98 ± 0.09	2.81 ± 0.07	0.04	0.910
Ammonia N (mg/dl)	13.07 ± 0.46	12.60 ± 0.80	12.60 ± 0.80	12.13 ± 1.68	13.67 ± 0.54	11.90 ± 1.212	11.83 ± 0.46	0.331	0.814

Means with different superscripts a, b, c, d and e in the same row differ significantly (*P* < 0.05)

## Discussion

### Chemical composition of ML

ML assists as a healthy and affordable source of micronutrients and proteins. It helps to alleviate the feeding trouble and act as alternative source for ruminants with high biological value. In this study, enhancement in OM and CP with ML addition were observed. Kholif et al. (2019) revealed that ML extract supplementation in Nubian goats enhanced the digestibility of OM, dry matter and NDF. Fadiyimu et al. (2010) also revealed that feeding concentrate along with ML (25%) significantly enhanced the intake of CP, DM, weight gain and nutrient digestibility in sheep. This may be due the production of phenolics, tannins and saponins by ML which can be used as energy sources in low amounts by rumen microbes during rumen fermentation (Bodas et al. 2012). CP fraction of ML was 264, 435 g/kg DM (Gupta et al. 1989; Makkar and Becker 1996). Sultana et al. (2015) reported that ML feed (100%) significantly improved the CP and NDF content in Bengal goats.

### Secondary metabolites in ML

Secondary metabolites found in ML include tannins, flavonoids, phenolics and saponins may also contribute to improvements in nutrient digestibility. Rumen microbes can utilize them as energy sources without negatively affecting rumen fermentation (Abdel-Raheem and Hassan 2021). They also have antibacterial and protozoal effects that help to reduce methane production while increasing acetate production, which improves carbohydrate digestion in ruminants (Parra-Garcia et al. 2019). SM also have an effect on ruminal cellulolytic and ammonia producing bacteria and limit the production of gases required for methanogenesis (Goel and Makkar 2012; Kholif and Olafadehan 2021).

### Mineral profile of different feeds

Mineral content varied as the feed available. Supplementation of plant derived feed having appropriate nourishing sources and one of the finest methods to improve the nutritional status in ruminants (Abou-Elkhair et al. 2020). ML are appropriate for animal feed not only for nutrients it also having low quantities of antinutrients. In this study magnesium and iron content were higher in ML as compared to berseem and concentrate mixture. Many plants are deficient in minerals like iron but ML contains calcium, phosphorus, iron, magnesium and zinc about 24,700, 4,400, 318.81, 190 and 22.05 (mg/kg) on dry matter basis (Teixeira et al. 2014). Calcium is the utmost plentiful mineral vital for the animal body involved in skeleton and teeth formation. Keeping the optimum level of minerals such as calcium and phosphorus is crucial for normal functioning of the body. Trace minerals also play an important function for animal body and required in small amount such as zinc, iron, manganese and copper which involved in tissue repairing, protein metabolism, improved immune status and had positive outcome on milk production of animals (Brisibe et al. 2009).

### Gas production and rumen fermentation parameters

Animal feed composition having critical aspect to control the methane emission. Recently ruminant methane reduction approaches involved the addition of some inhibitors such as chemical, biological and natural animal feed to inhibit the growth of methanogenic microbes in gut of animals. ML are effective methanogen inhibitors and thus considered alternatives for rumen fermentation pathways. In this study increase in total gas production was observed upto 10% level. Gas production is mainly due to liberation of acetate, propionate and butyrate by the fermentation of carbohydrates. In the present study it is revealed that as the roughage contents increased methane production also augmented, but addition of ML reduces the methane production at 0, 5, 10, 15, 20, 30 and 40% levels. This might be due to the presence of ɑ-linolenic, tannins and saponins in ML (Machmüller et al. 2000). Presence of tannins and phenolics had antimicrobial effects which can be a main cause for methane reduction (Goel and Makkar 2012). Reduction in methane (17%) was also observed in ML treated ruminants over soyabean meal (Soliva et al. 2015). Supplementation of ML by replacing soyabean meal significantly reduces methane production, ammonia-N in steers and goats, but increased the production of CO_2_ reported by Elghandour et al. (2017a, b). ML feed decreased enteric methane emission and increased milk production in dairy cows as reported by Bashar et al. (2020). ML feeding may reduce the energy loss including methane and urinary nitrogen without having an effect on beef cattle production (Sultana et al. 2021).

Higher ruminal digestibility of fibers and other constituents in ML reportedly contributes to its considerably high energy concentration. Dey et al. (2014) also reported increased in the TDMD and TOMD contents on supplementation of *M. oleifera* leaves to wheat straw. Supplementation of *M. oleifera* improve digestibility, sustained outstanding situation and confirm better feeding value (Cohen-Zinder et al. 2016). Therefore, improvement in TDMD and TDMO in the present study might be due to higher degradability of *Moringa* leaves as both the parameters improved with incremental levels of concentrate replacement with ML. Li et al. (2019) reported that ML diet can enhance nutrient intake, nutritional digestibility and rumen fermentation in dairy Holsteins cows. Aregheore (2020) reported that ML supplementation (20%) in growing goats improved digestibility and weight gain.

The increasing level of ML in the experimental did not affect ammonical nitrogen concentration in any of the TMR. Ammonia-N ratio of ruminal in this study reached from 12.02 to 13.14 (mg/dl). This could be due to the total dietary nitrogen level was at par (iso-nitrogenous) or with a small difference, and thus nitrogen degradation in the rumen occurred in a similar fashion among the R:C ratio or within the same ratio in different level of ML replacement. Elghandour et al. (2017a, b) reported that ML supplementation decreased the ruminal ammonia-N and protozoal population. Application of ML decreased ruminal ammonia-N due to presence of tannins and phenols help retain dietary proteins and slow down the degradability of rumen proteins (Kholif et al. 2015, 2016). Rumen protozoa are thought to be the primary source of rumen ammonia due to bacterial protein consumption and proteolysis (Bhatta et al. 2012). Reduction in ammonia-N also may be due to the decrease in the protozoal and bacterial population which involved in degradation of proteins in ruminants (Newbold et al. 2004). ML may play a function in limiting ammonia by reducing ruminal protein breakdown and deamination and as well as rumen ammonia. Higher VFA and lower ruminal ammonia during ML feed showed increased in consumption of dietary nitrogen (Babiker et al. 2017). Increase in propionate production also represents a change in rumen fermentation to reduce methane emission (Polyorach et al. 2014). *Moringa* leaves silage increased the total gas production, acetate, propionate while reduced the ruminal protozoa population and methane production (Morsy et al. 2022).

PF which is the ratio of in vitro substrate truly digested to gas volume (Blümmel et al. 1997) theoretically varies from 2.75 to 4.41 the values of PF of all the three groups with ML were within the normal range indicating proper portioning of nutrient for microbial mass production. The increase in MBP (mg) in the current study might be due to higher fraction of CP, in concurrence with greater ruminal degradability of ML protein (Makkar and Becker 1997). It might also be due to the improvement of the rumen microbiome and stimulation of fermentation process by the fermentable N and available carbohydrates supplied by *M. oleifera* leaves. ML supplementation altered ruminal fermentation and reduced in vitro greenhouse gases production (Kholif et al. 2022). Present study revealed that supplementation of ML improved protein content, digestibility rate, microbial biomass and partial fraction and reduces methane gas emission.

*Moringa oleifera* leaves can be used as a protein basis in diet of goats under in vitro conditions. Supplementation of ML improved the nutrient digestibility, rumen fermentation parameters and corresponding decrease in methane production. Consequently, it can be concluded that *M. oleifera* leaf powder can be replaced up to 10–20% of concentrate as a protein source and for methane mitigation in ruminants. Still, further study on different animals with different concentration of ML is needed to validate/expand the results under in vitro and in vivo conditions.

## Data Availability

All data are presented in tables and figures within this manuscript.

## Abbreviations

ML: Moringa leaves
TDMD: True dry matter digestibility
PF: Partial fraction
CP: Crude protein
EE: Ether extract
OM: Organic matter
MBP: Microbial biomass
TMR: Total mixed ration
